# Current developments in 3D printing technology for orthopedic trauma: A review

**DOI:** 10.1097/MD.0000000000041946

**Published:** 2025-03-21

**Authors:** Kun Ling, Wenzhu Wang, Jie Liu

**Affiliations:** a Department of Emergency Medicine, West China Hospital, Sichuan University/West China School of Nursing, Sichuan University, Chengdu, China; b Disaster Medical Center, Sichuan University, Chengdu, China; c Nursing Key Laboratory of Sichuan Province, Chengdu, China.

**Keywords:** 3D printing, additive manufacturing, orthopedic trauma, surgical planning

## Abstract

Three-dimensional (3D) printing technology has emerged as a revolutionary tool in orthopedic trauma surgery, offering unprecedented opportunities for personalized patient care. This comprehensive review explores the current developments and applications of 3D printing in orthopedic trauma, highlighting its potential to address complex surgical challenges. We provide an in-depth analysis of various 3D printing technologies applicable to orthopedic surgery, including vat photopolymerization, material extrusion, powder bed fusion, and sheet lamination. The review examines the use of 3D printing in preoperative planning, surgical simulation, and the creation of patient-specific implants and surgical guides. We discuss applications across different anatomical regions, including upper limb, lower limb, and pelvic and spinal trauma. Evidence from recent studies demonstrates that 3D printing-assisted surgeries can lead to reduced operative times, decreased blood loss, improved fracture reduction quality, and potentially better clinical outcomes. This review synthesizes the latest research and clinical experiences, providing insights into the current state of 3D printing in orthopedic trauma and its future perspectives. As the technology continues to evolve, 3D printing holds promise for increasingly personalized and effective treatments in orthopedic trauma care, potentially transforming surgical practices and improving patient outcomes.

## 1. Introduction

Orthopedic trauma presents unique challenges in surgical management, often requiring precise anatomical reduction and fixation to restore function and prevent long-term complications. The complexity of fracture patterns, particularly in areas such as the pelvis, acetabulum, and periarticular regions, demands innovative approaches to improve surgical outcomes.^[1]^ In recent years, three-dimensional (3D) printing technology has emerged as a transformative tool in orthopedic trauma surgery, offering new possibilities for patient-specific treatments, surgical planning, and implant design.^[2,3]^ As highlighted by Xu et al, this technology shows particular promise for repairing large-area irregular bone defects, where conventional treatment methods often fall short. 3D printing, also known as additive manufacturing, encompasses a range of technologies that create three-dimensional objects by depositing materials layer by layer.^[4]^ These technologies include vat photopolymerization, material extrusion, powder bed fusion, and sheet lamination, each with its unique advantages and applications in medical contexts. The ability to create complex, customized structures has opened up new avenues for addressing the challenges faced by orthopedic surgeons in trauma cases.

The application of 3D printing in orthopedic trauma spans various aspects of patient care. Preoperatively, 3D-printed anatomical models enable surgeons to better understand fracture morphology, plan surgical approaches, and simulate procedures.^[5]^ Intraoperatively, patient-specific surgical guides and customized implants can enhance the precision of fracture reduction and fixation.^[6]^ Recent advances in height profile control, as demonstrated by Wu et al, have significantly improved the geometric integrity of 3D printed components, enabling more precise fabrication of anatomical models.^[7]^ Furthermore, 3D printing facilitates the creation of patient-specific implants that can better match the unique anatomy of each individual, potentially improving functional outcomes.^[8]^ The integration of artificial intelligence with 3D bioprinting, as proposed by Lee, offers promising pathways for standardizing and automating the fabrication of complex biological structures, potentially revolutionizing the production of customized orthopedic implants.^[9]^ Recent studies have demonstrated the benefits of 3D printing technology in various orthopedic trauma scenarios. For instance, in the treatment of acetabular fractures, 3D printing-assisted surgery has been shown to reduce operative time, decrease blood loss, and improve the quality of fracture reduction compared to traditional techniques.^[10]^ Quantitative evidence further substantiates these claims: Yang et al^[11]^ documented a 32% reduction in blood loss and 15% higher functional scores in 3D-assisted elbow fracture surgeries; Papotto et al’s^[12]^ systematic review of acetabular fractures reported average reductions of 25% in operative time and 30% in blood loss; while Tomaževič et al^[13]^ demonstrated statistically significant improvement in fracture reduction accuracy (*P* < .001) with patient-specific 3D printed implants. Similarly, in complex periarticular fractures, the use of 3D-printed models and surgical guides has led to more accurate implant placement and improved surgical outcomes.^[14]^ Improved height difference-based models, as developed by Wu and Chiu, have further enhanced the precision of drop-on-demand 3D printing, allowing for more accurate reproduction of complex bone geometries.^[15,16]^ Despite the promising results, the integration of 3D printing technology in orthopedic trauma surgery faces several challenges, including regulatory hurdles, cost considerations, and the need for specialized training.^[17]^ Additionally, while short-term outcomes have been encouraging, long-term follow-up studies are necessary to fully evaluate the efficacy of 3D printing-assisted surgeries.^[18]^

Recent reviews have made significant contributions to understanding 3D printing applications in orthopedic trauma, yet with varying scopes and emphases. Neijhoft and IJpma (2024)^[19]^ documented advances in 3D printing for tibial plateau fractures, foot and ankle fractures, and distal radius corrective osteotomies, highlighting its educational value, but primarily focused on selected fracture types without comprehensive coverage of all anatomical regions in orthopedic trauma. Duan et al (2021)^[20]^ explored 3D printing applications in orthopedic treatment, including preoperative planning, surgical guides, personalized implants, and customized prostheses, detailing eleven clinical cases, yet with limited discussion on the fundamental principles and technical specifications of different 3D printing technologies. Wixted et al (2021)^[21]^ provided a comprehensive review of 3D printing in orthopedic surgery, encompassing anatomical models, prosthetics, non-custom implants, and patient-specific instrumentation, with a brief introduction to bioprinting’s future potential, but lacking robust quantitative data analysis and cost-effectiveness evaluation. While existing literature has elucidated the value of 3D printing in orthopedic trauma from different perspectives, there remains insufficient in-depth discussion of technological limitations, inadequate exploration of emerging technologies such as 4D printing, and a scarcity of comprehensive clinical application data or systematic application guidelines.

This review aims to address these gaps by providing a more comprehensive and in-depth analysis of 3D printing technology in orthopedic trauma. We present detailed principles and characteristics of various 3D printing technologies, systematically analyze their applications and clinical outcomes in upper limb, lower limb, and pelvic/spinal trauma, and synthesize quantifiable benefits from recent studies. Through integrating the latest research, we elucidate current developments and future directions of 3D printing in orthopedic trauma care, identifying opportunities for continued innovation and areas requiring further investigation.

## 2. Overview of 3D printing technology

**Figure 1.**
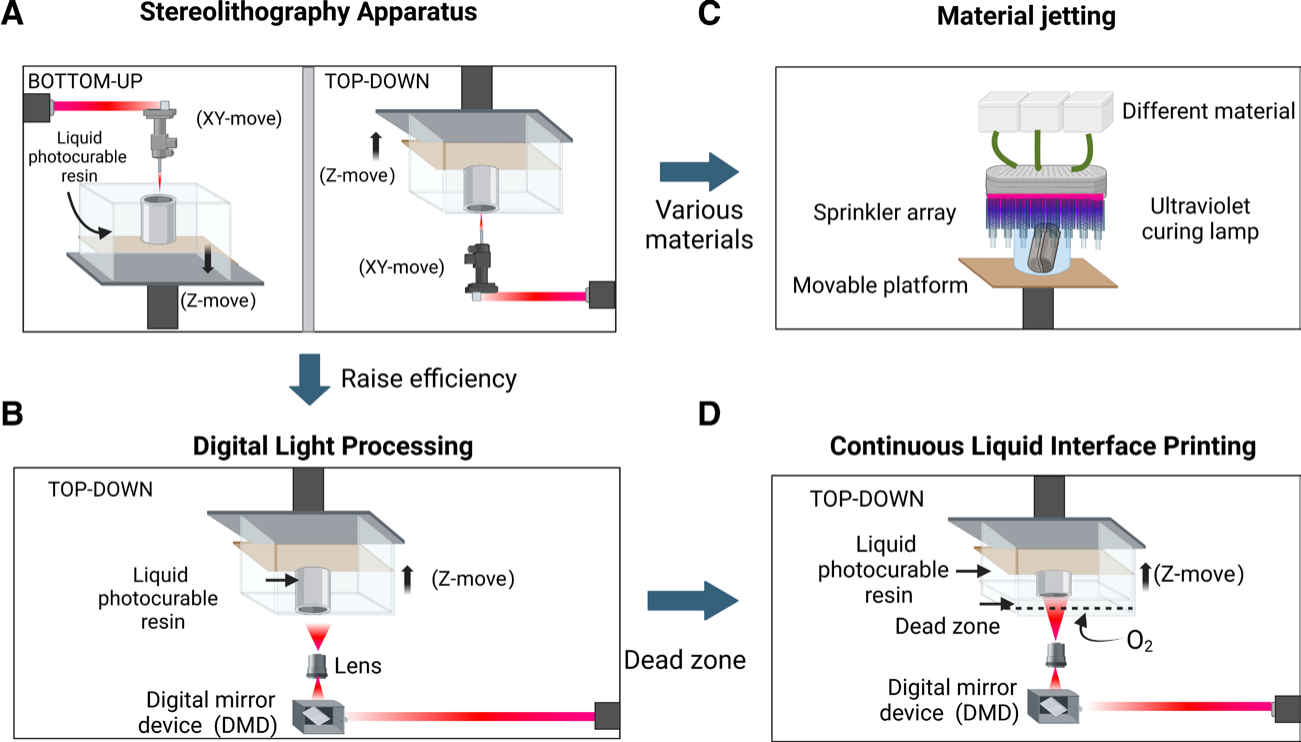
Stereolithography in 3D printing technology. (A) Scheme of bottom-up and top-down stereolithography setups, the laser beam selectively scans the liquid photosensitive resin according to the cross-sectional data of the object being printed. (B) Digital Light Processing, instead of point-by-point scanning with a laser, the layer onto the surface of the photosensitive resin was applied. (D) Continuous Liquid Interface Printing, comparing DLP an oxygen-permeable window was incorporates at the bottom of the resin vat to prevents the cured object from adhering to the window, greatly reducing the risk of print failures and allowing for continuous upward movement of the build platform. (C) Material Jetting, MJ technology employs an array of print heads to deposit thousands of photopolymer droplets onto the build platform, which are then cured using UV light.

3D printing, also known as additive manufacturing, has emerged as a transformative technology with numerous applications in various fields, including orthopedic trauma. The ability to create complex, customized structures layer by layer has opened up new possibilities for patient-specific treatments, surgical planning, and implant design in orthopedic trauma.^[22]^ As the technology continues to advance, it is expected to play an increasingly important role in addressing the unique challenges faced by orthopedic surgeons and improving patient outcomes.^[23]^ The following sections provide an overview of the main 3D printing technologies, including vat photopolymerization, material extrusion, powder bed fusion, and sheet lamination (Fig. 1; Table 1).

**Table 1:** Comparison of 3D printing technologies: techniques, materials, advantages, and limitations

3D printing technology	Specific techniques	Materials	Advantages	Limitations
Vat Photopolymerization	SLA	Photosensitive resins (acrylates, epoxies)	High accuracy; Good surface quality	Limited materials;
	DLP	Photosensitive resins (acrylates, epoxies)	High accuracy; Fast printing	Limited materials; High equipment cost;
	CLIP	Photosensitive resins (acrylates)	Fast printing	Special materials required
	MJ	Photosensitive resins (acrylates)	Multi-material printing	High usage cost
	TPP	Photosensitive resins (acrylates)	Nanoscale resolution	Low efficiency; Special materials required
Material Extrusion	FDM	Thermoplastics (ABS, PLA, PC, PA.)	Low cost; Multi-material printing;	Poor surface quality; Low accuracy; Low efficiency
	DIW	Plastics, ceramics, living cells, food	Wide range of materials	Poor surface quality; Low accuracy; Special post-processing
Powder Bed Fusion	SLM	Various metals	High accuracy; Good mechanical properties;	High cost; Internal stress; Special post-processing
	SLS	Various metals, thermoplastics	Reduced support material	High cost; Rough surface
	EBM	Various metals	Good mechanical properties; High efficiency;	High cost; Rough surface; Low accuracy
Binder Jetting	3D Printing	Polymers, metals, ceramics (powdered)	Multi-material and color printing; Lower cost than other powder-based technologies;	Poor density and mechanical strength
Sheet Lamination	LOM	Paper, plastic films, composite sheets	Low cost; High efficiency;	Poor mechanical properties; Limited structural complexity

### 2.1. Vat photopolymerization

Vat photopolymerization is an additive manufacturing process that utilizes a liquid photopolymer resin, which is selectively cured by a light source in a layer-by-layer fashion to create three-dimensional objects.^[4]^ This process is known for its high accuracy, smooth surface finish, and ability to produce complex geometries.^[24]^ Vat photopolymerization encompasses several specific technologies, including stereolithography, digital light processing, material jetting and two-photon polymerization.^[25]^ (Fig. 1).

#### 2.1.1. Stereolithography

Stereolithography is a pioneering vat photopolymerization technique based on the principle of photopolymerization, where liquid photosensitive resins solidify upon exposure to light of a specific wavelength.^[26]^ This concept of layer-by-layer manufacturing using photopolymerization was first proposed by Kodama et al in 1981. In 1984, Hull developed the first commercialized system based on this principle, known as stereolithography apparatus (SLA). In SLA, an ultraviolet laser beam is used as the light source for curing the resin. The laser beam is controlled by a scanning galvanometer and selectively scans the surface or bottom of the liquid photosensitive resin according to the cross-sectional data of the object being printed.^[27]^ The scanned area of the resin layer undergoes a polymerization reaction and solidifies, forming a single thin layer of the object. The build platform then moves downward or upward by a distance equal to the layer thickness, allowing a new layer of liquid resin to cover the previously cured layer.^[28]^ This process is repeated until the entire object is built up layer by layer, resulting in a complete three-dimensional object.^[29]^ SLA is capable of producing high-resolution parts with excellent surface quality, achieving resolutions between 25 and 100 μm.^[30]^ Current commercial SLA systems can produce objects with maximum dimensions of up to 800 mm × 330 mm × 400 mm in a single build.^[31]^ SLA is widely used in various applications, including medical modeling, dental restorations, and rapid prototyping.^[32,33]^

#### 2.1.2. Digital light processing

Digital Light Processing (DLP) is another vat photopolymerization technique that was developed to improve the efficiency of the SLA process. While DLP shares the same basic principles, materials, and achieves similar resolution as SLA, it differs in its approach to curing each layer.^[34]^ Instead of point-by-point scanning with a laser, DLP uses a digital micromirror device to project the entire cross-sectional image of each layer onto the surface of the photosensitive resin.^[35]^ This approach significantly increases the printing speed compared to SLA, as each layer is cured in a single step, regardless of the number of objects being printed simultaneously.^[36,37]^ The total printing time for DLP is solely dependent on the height of the object being fabricated.^[38]^ In 2015, DeSimone et al^[39,40]^ and his team introduced an improved version of DLP technology called Continuous Liquid Interface Printing. Continuous Liquid Interface Printing follows the same basic principles as DLP but incorporates an oxygen-permeable window at the bottom of the resin vat, in addition to the transparent window that allows UV light to pass through. By controlling the amount of oxygen passing through the window, a thin layer (20–30 μm) of uncured resin, known as the “dead zone,” is maintained just above the window.^[41]^ This liquid dead zone prevents the cured object from adhering to the window, greatly reducing the risk of print failures and allowing for continuous upward movement of the build platform.^[42]^ This innovation dramatically increases printing efficiency, with reported printing speeds of up to 30 cm/h at Z-axis resolutions below 100 μm and over 100 cm/h for low-resolution objects.^[39]^

#### 2.1.3. Material jetting

Material Jetting (MJ) is another important branch of vat photopolymerization technologies that differs from the aforementioned techniques in its material deposition method. Instead of using a vat filled with photosensitive resin, MJ technology employs an array of print heads to deposit thousands of photopolymer droplets onto the build platform, which are then cured using UV light.^[43]^ This technology, also known as PolyJet, was first patented and commercialized by Objet, an Israeli company, in 2000. A typical MJ printer consists of material containers, a movable build platform, and a carriage equipped with UV curing lamps and an array of print heads. Prior to printing, various types and colors of photosensitive resins are loaded into separate material containers and heated to achieve the desired viscosity.^[44]^ During the printing process, the array of print heads moves along the X-axis over the build platform, selectively jetting droplets according to the cross-sectional data of each layer of the object being printed. The UV lamps immediately cure the jetted droplets.^[44]^ The presence of multiple print heads enables the simultaneous printing of different colors and materials, making MJ technology particularly suitable for creating realistic, full-color models.^[45,46]^ One of the key advantages of MJ technology is its ability to produce high-resolution, multi-material objects with smooth surfaces and fine details.^[47]^ This capability has made MJ a popular choice for various applications, including rapid prototyping, dental models, and medical devices.^[48]^ However, the technology also has some limitations, such as relatively high material costs and the need for post-processing to remove support structures.^[49]^

#### 2.1.4. Two-photon polymerization, TPP

While SLA, DLP, and MJ technologies rely on single-photon polymerization, some materials exhibit a special energy level transition mode that allows for the simultaneous absorption of two photons, known as the “two-photon absorption effect.”^[50]^ The conditions for two-photon absorption are stringent, requiring the use of special photosensitive resins and a highly focused laser beam with sufficient irradiance at its center to ensure the simultaneous absorption of two photons by the resin, triggering polymerization.^[51]^ This phenomenon enables the precise control of resin solidification within the nanoscale range at the focal point of the laser. When combined with a nanoscale precision motion platform, this forms the basis of two-photon polymerization (TPP) technology, which is capable of printing ultra-fine structures.^[52]^ TPP technology offers significant advantages in the field of micro- and nanoscale fabrication. However, due to the high cost of equipment and the complex nature of the fabrication process, TPP is currently primarily used in scientific research.^[53]^

### 2.2. Material extrusion

**Figure 2.**
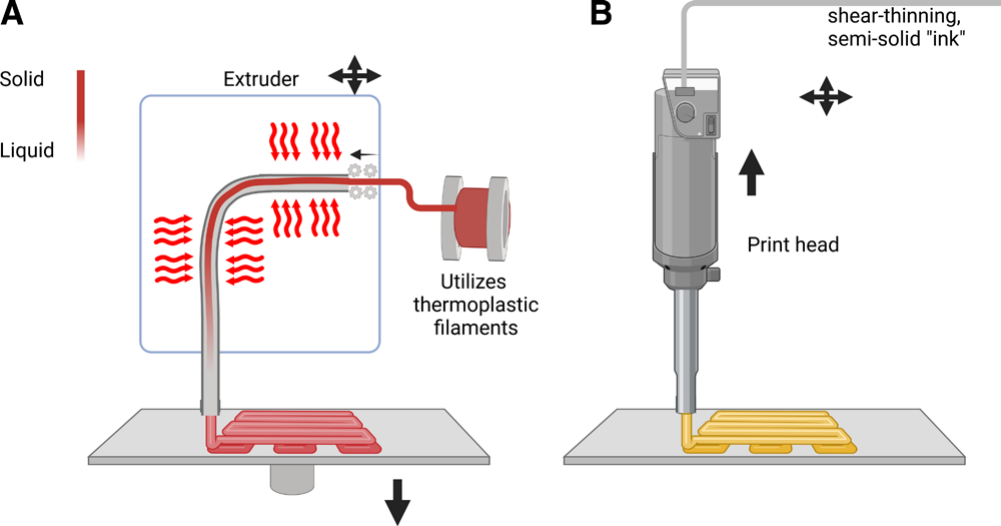
Material extrusion in 3D printing technology. (A) Fused deposition modeling process (FDM). The filament is heated to a semi-molten state inside the print head, which then moves along the contours and infill paths of the object’s cross-section, continuously extruding the material. The extruded material rapidly solidifies upon cooling and bonds with the surrounding material. Once a layer is completed, the build platform lowers by one layer thickness. (B) Direct Ink Writing (DIW). This technique involves extruding a shear-thinning, semi-solid “ink” material through a nozzle and stacking the layers to construct a pre-designed 3D structure by print head which enabling X, Y, and Z movement without the need for a movable build platform.

Material extrusion is a widely used 3D printing technology that includes two main techniques: Fused Deposition Modeling (FDM) and Direct Ink Writing (DIW) (Fig. 2).

#### 2.2.1. Fused deposition modeling

FDM is a widely used material extrusion 3D printing technique that utilizes thermoplastic filaments such as polylactic acid, acrylonitrile butadiene styrene, and polycarbonate as raw materials.^[54,55]^ The filament is heated to a semi-molten state inside the print head, which then moves along the contours and infill paths of the object’s cross-section, continuously extruding the material. The extruded material rapidly solidifies upon cooling and bonds with the surrounding material. Once a layer is completed, the build platform lowers by one layer thickness, and the process is repeated until the entire object is formed.^[56]^ FDM’s simplicity and low maintenance costs have made it one of the most popular 3D printing technologies. Commercial FDM systems can achieve print resolutions of 100 to 150 μm and build volumes up to 1005 mm × 1005 mm × 1005 mm.^[57]^ Dual or multiple print heads enable the simultaneous printing of different materials, allowing for multi-color printing. However, FDM’s main limitation is its relatively low printing speed, especially when fabricating high-resolution or large-scale objects.^[58]^

#### 2.2.2. Direct ink writing

DIW, also known as Robocasting, is another material extrusion technique that differs from FDM in its print head design.^[59]^ In DIW, the print head is directly connected to a robotic arm, enabling X, Y, and Z movement without the need for a movable build platform.^[60]^ The technique involves extruding a shear-thinning, semi-solid “ink” material through a nozzle and stacking the layers to construct a pre-designed 3D structure.^[61]^ DIW’s main advantage lies in its versatility in terms of printable materials, including conductive pastes, elastomers, and hydrogels, which possess rheological properties (e.g., viscoelasticity, shear-thinning, yield stress) that facilitate the 3D printing process.^[62]^

### 2.3. Powder bed fusion

**Figure 3.**
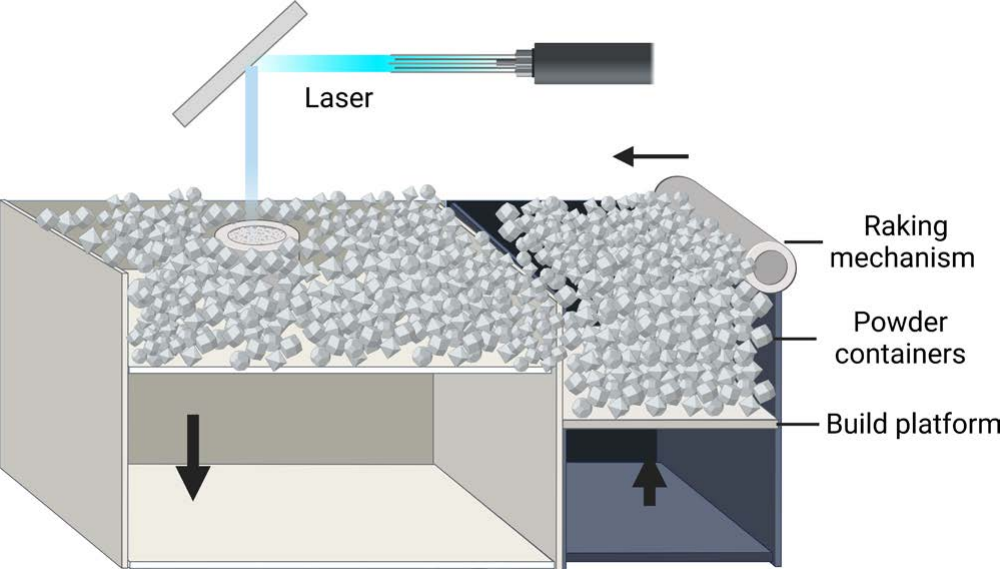
Powder bed fusion in 3D printing technology. Powder Bed Fusion. According to different sources of heat energy, powder bed fusion can be divided into laser- and electron beam-based powder bed fusion. Selective laser melting (SLM) is the most classic and commonly used molding technology.

Powder bed fusion (PBF) is a category of additive manufacturing processes that primarily encompasses the production of metal and polymer parts. The three most prominent PBF techniques are selective laser melting, electron beam melting, and selective laser sintering^[63]^ (Fig. 3).

#### 2.3.1. Selective laser melting

Selective laser melting (SLM), also known as laser powder bed fusion, utilizes a high-energy laser beam to selectively melt and fuse metal powder particles layer by layer.^[64]^ The process begins with a thin layer of metal powder spread evenly onto a build platform using a recoater blade or roller. A laser beam then scans the powder bed, melting and fusing the particles in specific areas according to the cross-sectional data of the current layer. After completing a layer, the build platform lowers by one layer thickness, and a new layer of powder is deposited on top. This process repeats until the entire part is fabricated.^[65]^

SLM typically operates in an inert gas environment (argon or nitrogen) to prevent oxidation of the metal powder.^[66]^ The laser beam used in SLM has a smaller spot size compared to the electron beam in electron beam melting (EBM), enabling the production of finer features and more complex geometries.^[67]^ However, the laser energy is partially reflected by the metal powder, resulting in lower energy utilization compared to EBM.^[68]^ Additionally, SLM parts often experience rapid cooling, leading to internal stress concentrations that may require post-process heat treatment to alleviate residual stresses.^[69]^

#### 2.3.2. Electron beam melting

EBM is another PBF technique that uses a high-energy electron beam to melt and fuse metal powder particles.^[70]^ The process is similar to SLM, with the main difference being the energy source. EBM operates in a high vacuum environment, which prevents oxidation and allows for the processing of reactive materials such as titanium alloys.^[71]^

One of the key advantages of EBM is its higher energy utilization compared to SLM, as the electron beam is not significantly reflected by the metal powder.^[72]^ This makes EBM particularly suitable for processing materials with high thermal conductivity, high-temperature alloys, and high-melting-point metals.^[73]^ Additionally, EBM preheats each layer of powder using a defocused electron beam, maintaining the part at an elevated temperature (600–1200°C) during the build process. This preheating significantly reduces residual stresses in the fabricated parts, often eliminating the need for post-process heat treatment.^[74]^

#### 2.3.3. Selective laser sintering, SLS

Selective Laser Sintering (SLS) is another prominent powder bed fusion technique that utilizes a laser to sinter powdered materials, such as polymers, metals, or ceramics, into a solid 3D object.^[75]^ Unlike SLM and EBM, which fully melt the metal powder, SLS works by heating the powder to a temperature just below its melting point, causing the particles to fuse together through solid-state diffusion.^[76]^ In the SLS process, a thin layer of powder is spread evenly across the build platform. A CO2 laser then selectively scans the powder bed, sintering the particles in specific areas according to the cross-sectional data of the current layer. After completing a layer, the build platform lowers by one layer thickness, and a new layer of powder is deposited on top. This process is repeated until the entire object is fabricated.^[77]^ One of the key advantages of SLS is its ability to process a wide range of materials, including polymers (e.g., nylon, polystyrene), metals (e.g., stainless steel, titanium), and ceramics (e.g., alumina, zirconia).^[78]^ Additionally, SLS does not require support structures for overhanging features, as the unsintered powder acts as a natural support, simplifying post-processing and enabling the creation of more complex geometries.^[79]^ However, SLS parts typically have a rougher surface finish and lower dimensional accuracy compared to SLM and EBM parts, due to the partial melting of the powder particles.^[80]^ Post-processing techniques, such as sanding, polishing, or coating, may be necessary to achieve the desired surface quality and mechanical properties.^[81]^

### 2.4. Sheet lamination

**Figure 4.**
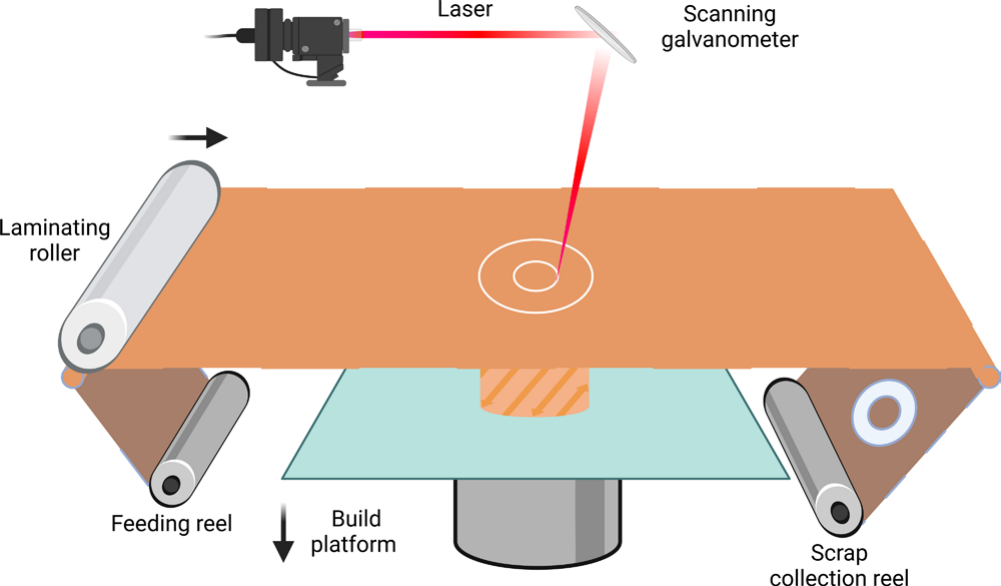
Sheet lamination in 3D printing technology. Laminated Object Manufacturing (LOM). Using sheets such as paper, plastic film or composite materials as the basis. The laser beam is used as an energy source to cut the sheet layer by layer, and the hot pressing device stacks and bonds the sheet layer by layer to finally form a three-dimensional component.

Sheet lamination is a family of additive manufacturing processes that involve the bonding of sheets of material together to form a 3D object. The main sheet lamination technique used in additive manufacturing is Laminated Object Manufacturing (LOM).^[82]^ LOM is a sheet lamination process that uses adhesive-coated paper, plastic, or metal sheets as the raw material. In LOM, a laser or knife is used to cut the outline of each layer of the object based on the (Computer-Aided Design) data. The excess material is then removed, and a new sheet is bonded on top of the previous layer using heat and pressure. This process is repeated until the entire object is fabricated.^[82]^ One of the advantages of LOM is its ability to create large parts quickly and at a low cost compared to other additive manufacturing techniques. Additionally, LOM can use a wide range of materials, including paper, plastic, and metal foils, making it suitable for various applications.^[83]^ However, LOM-produced parts often have a rough surface finish due to the layer-by-layer nature of the process and the presence of visible seams between the sheets. Post-processing techniques, such as sanding or filling, may be required to achieve a smoother surfac. Moreover, the mechanical properties of LOM parts are often anisotropic and may be limited by the strength of the adhesive used to bond the layers together (Fig. 4).

## 3. Challenges in treating complex traumatic fractures

Complex traumatic fractures pose significant challenges for orthopedic surgeons, often requiring surgical intervention to restore proper alignment, stability, and function of the affected bones.^[84]^ The complexity of these fractures is compounded by the wide range of fracture patterns and individual variations in patient anatomy. For example, in a study of 100 patients with trimalleolar fractures, Xiao et al^[5]^ found that the fracture patterns varied considerably, with 37 different types identified. This diversity makes it difficult for surgeons to develop standardized treatment plans, as each fracture case requires a tailored surgical approach.

Moreover, traditional surgical methods often rely on generic implants and instrumentation, which may not accommodate the unique anatomical variations of each patient.^[2]^ This lack of flexibility and precision can result in suboptimal fracture reduction and fixation, leading to poor outcomes. In a study on three fracture models, Qiao et al^[85]^ demonstrated that their novel 3D printed customized external fixator (Q-Fixator) achieved excellent reduction results, with an average rotational deformity of 1.21°, angulation of 1.84°, and lateral displacement of 2.22 mm. These results suggest that patient-specific implants, designed to match individual fracture patterns and anatomy, may help overcome the limitations of generic fixators and improve surgical outcomes for complex fractures.

The limitations of current surgical approaches are further exacerbated by the high surgical complexity and complication risks associated with complex traumatic fractures. These injuries often require extensive surgical exposure, prolonged operative time, and advanced surgical skills, which contribute to an increased risk of complications, such as infection, implant failure, and nonunion. In a retrospective study of 283 patients with complex tibial plateau fractures, Jansen et al^[86]^ reported an average surgical time of 130 minutes, with a deep infection rate of 5.7% and a nonunion rate of 7.1%. These findings highlight the need for innovative surgical techniques and technologies that can simplify procedures, reduce operative time, and minimize complications.

Given these challenges, there is a clear need for innovative solutions that can improve the personalization, precision, and safety of surgical treatments for complex traumatic fractures. 3D printing technology has emerged as a promising tool to address these limitations, enabling the creation of patient-specific implants, guides, and models that can optimize surgical planning and execution.

## 4. Applications of 3D printing in orthopedic trauma

### 4.1. Upper limb trauma

#### 4.1.1. Acromion and clavicle

Beliën et al^[87]^ developed a novel 3D printing-assisted technique for treating OS acromiale and acromial fractures. They created a 3D-printed model of the patient’s acromion based on CT scan data and pre-bent an osteosynthesis plate to match the model’s anatomical shape. During surgery, the pre-contoured plate was fixed onto the acromion, and osteotomy was performed for OS acromiale cases. This patient-specific approach reduced surgical time, improved surgeon-patient communication, and showed complete healing of fractures and non-unions in postoperative follow-up. Jeong et al and Kim et al^[88,89]^ utilized 3D printing technology to enhance minimally invasive plate osteosynthesis for midshaft clavicular fractures. By creating 3D-printed models of the fractured and contralateral clavicles, they accurately prebent locking plates to fit the patient’s unique anatomy, facilitating precise fracture reduction and minimizing soft tissue damage. These techniques demonstrated effective bone union and reduced operative complications (Table 2).

**Table 2:** Applications of 3D printing in upper limb trauma

Application	Technique	Advantages	Outcomes	Ref
Acromion & Clavicle	3D-printed models for pre-bending plates	Reduced surgical time, improved communication	Complete healing of fractures and non-unions	[87]
	3D-printed models for MIPO	Accurate prebending of plates	Effective bone union, reduced complications	[88,89]
Proximal Humerus	3D-printed fracture models for planning	Reduced surgery duration, less blood loss	Improved surgical efficiency	[90]
Distal Humerus	Patient-specific 3D-printed plates	Reduced operative time	Superior clinical outcomes	[91]
	Customized osteotomy guides	Precise osteotomy	Improved elbow function, deformity correction	[11]
Distal Radius	3D-printed corrective osteotomy	Restored anatomical parameters	Improved motion, strength	[92]
Hand	Preoperative planning for thumb reconstruction	Guided bone cutting, flap shaping	Improved function, patient satisfaction	[93]
Vascularized Bone Transfer	3D models for bone flap contouring	Precise intraoperative design	Enhanced reconstruction	[94]
Shoulder Arthroplasty	Customized titanium glenoid implant	Matched bone defect morphology	Pain relief, improved function	[95]

#### 4.1.2. Proximal humerus

You et al^[90]^ investigated the clinical potential of 3D printing technology in treating complicated proximal humeral fractures in elderly patients. The study compared a 3DPT (3D Printing Technology) group, which utilized 3D-printed fracture models for preoperative planning and surgical simulation, with a control group relying solely on thin-layer CT scans. The 3DPT group showed significant improvements in surgery duration, intraoperative blood loss, and fluoroscopy times, highlighting the efficacy of 3D printing in orthopedic trauma surgeries.

#### 4.1.3. Distal humerus

Shuang et al^[91]^ used 3D-printed patient-specific osteosynthesis plates for treating intercondylar humeral fractures. They reconstructed 3D fracture models based on CT data and fabricated individualized 3D-printed plates. The plates perfectly matched the fracture morphology, reducing operative time and achieving superior clinical outcomes compared to conventional methods. Zheng et al and Gemalmaz et al^[92,93]^ utilized 3D printing to create customized osteotomy guides for accurate correction of cubitus varus deformity. Using CT scans and mirror imaging, they designed and printed individualized osteotomy guides that facilitated precise intraoperative osteotomy and bone grafting. Follow-up results showed satisfactory deformity correction, improved elbow function, and the avoidance of lateral condyle prominence.

Yang et al^[11]^ evaluated the perioperative effect of 3D printing technology in treating complex elbow fractures. The 3D printing-assisted surgery group had significantly shorter surgical duration, lower intraoperative blood loss, and higher elbow function scores compared to the conventional group. The study also found polylactic acid to be a more suitable 3D printing material for surgical modeling than acrylonitrile butadiene styrene.

#### 4.1.4. Distal radius

de Muinck Keizer et al^[94]^ systematically evaluated the efficacy of 3D-printed corrective osteotomy for distal radius malunions. Compared with conventional 2D planning methods, 3D printing-assisted corrective osteotomy restored anatomical parameters to near-normal values in 96% of patients, significantly improving range of motion, forearm rotation, and grip strength, with a complication rate of 16%.

#### 4.1.5. Hand

Zhang et al^[95]^ reported the clinical application of 3D printing for preoperative planning of thumb reconstruction with toe transplantation. 3D-printed models of the injured and contralateral thumbs and feet were used to simulate and design the surgical plan, guiding intraoperative bone cutting and flap shaping. Postoperative results showed improved morphological parameters, opposing function, joint range of motion, and patient satisfaction.

#### 4.1.6. Vascularized bone transfer

Taylor et al^[96]^ explored surgeon-based design of 3D models using home 3D software and printing technology for vascularized bone transfer. The models facilitated intraoperative design and precise contouring of vascularized bone flaps, including medial femoral trochlea flaps, medial femoral condyle flaps, and free fibula osteocutaneous flaps, for various upper limb reconstructions.

#### 4.1.7. Shoulder arthroplasty

Stoffelen et al^[97]^ reported a case of using 3D printing to reconstruct a severely deficient glenoid in revision shoulder arthroplasty. A customized titanium glenoid implant was designed and fabricated based on CT data to match the bone defect morphology and provide adequate fixation. At 2.5 years follow-up, the patient’s pain was significantly relieved, with improved functional scores and no signs of implant loosening.

### 4.2. Lower limb trauma

The application of 3D printing technology in the management of lower limb trauma has garnered considerable interest in recent years. This innovative approach has been leveraged to tackle the various challenges associated with complex fractures, deformities, and ligament reconstructions. By harnessing the potential of 3D printing, surgeons can generate patient-specific models, guides, and implants, thereby enhancing surgical planning, precision, and outcomes (Table 3).

**Table 3:** Applications of 3D printing in lower limb trauma

Application	Technique	Advantages	Outcomes	Ref
Pelvic & Acetabular Fractures	3D models for planning	Enhanced fracture understanding	Better implant fit	[98]
	Patient-specific plates	Superior fit	Near-anatomical reduction	[99]
	Minimally invasive screw fixation	Reduced operative time	Comparable outcomes	[100]
	3D printing-assisted surgery	Reduced operative time, blood loss	Improved fracture reduction, clinical outcomes	[12]
	Patient-specific plates, guides	Improved reduction accuracy	Smaller displacement of fracture lines	[13]
	3D-printed plate templates	Reduced pre-contouring time	Time-efficient	[101]
	Custom-made metal plates	Better hip function, pain scores	Long-term benefits	[102]
Femoral & Tibial Fractures	Preoperative planning for femoral fractures	Accurate plate, screw positioning	Confirmed by CT	[103]
	3D cutting guides for osteotomies	Improved correction accuracy	Reduced surgical time	[104]
	3D planning for tibial fractures	Accurate fixation	Minimal deviations	[105,106]
Distal Tibial & Foot Fractures	3D models for planning	Enhanced fracture understanding	Favorable outcomes	[107]
	3D printing for talar neck fractures	Optimal screw placement	Stability, reduced complications	[108]
Ligament Reconstruction	Navigational templates for ankle ligaments	Precise reconstruction	Improved stability	[109]

#### 4.2.1. Pelvic and acetabular fractures

Pelvic and acetabular fractures are notoriously complex and challenging to manage due to their intricate anatomy and the necessity for accurate reduction and fixation. 3D printing has emerged as an invaluable tool for comprehending fracture patterns, devising surgical strategies, and optimizing implant placement. Numerous studies have highlighted the advantages of employing 3D printed models for preoperative planning and intraoperative guidance in the management of acetabular fractures. Hurson et al^[98]^ reported that 3D printed models significantly aided surgeons, especially novice surgeons, in grasping individual fracture anatomy. Maini et al^[99]^ discovered that patient-specific pre-contoured plates fabricated using 3D models yielded superior implant fit compared to intraoperatively contoured plates. Furthermore, Bagaria et al^[110]^ underscored the significance of 3D printing in achieving near-anatomical reduction in complex acetabular fractures.

Kim et al^[111]^ retrospectively analyzed their experience with 3D printed models in 14 cases of acetabular fractures and 10 cases of clavicular fractures. The models facilitated the understanding of pathoanatomy, planning of reduction clamp positioning, determination of screw entry sites and trajectories, and prebending of reconstruction plates. Additionally, the models enhanced resident training and precise positioning of percutaneous posterior column screws. In the context of pelvic fractures, Cai et al^[100]^ compared the outcomes of minimally invasive cannulated screw fixation with and without 3D printing assistance in 137 cases. The 3D printing group exhibited significantly reduced operative time and intraoperative fluoroscopy usage while achieving comparable reduction quality and functional outcomes to the control group. Wu et al^[112]^ assessed the accuracy and feasibility of 3D printing for the operative treatment of old pelvic fractures, finding a strong correlation between preoperative planning and postoperative radiographs. Zeng et al^[113]^ evaluated the efficacy of 3D printing-assisted internal fixation using a minimally invasive para-rectus approach in 38 cases of unstable pelvic fractures. The technique allowed for the rehearsal of plate positioning and screw trajectories on 3D printed models, resulting in accurate implant placement, minimal trauma, reduced blood loss, and excellent functional outcomes.

#### 4.2.2. Femoral and tibial fractures

3D printing has also found applications in the management of femoral and tibial fractures, particularly in cases involving complex fracture patterns, deformities, or the need for osteotomies. Lin et al^[103]^ utilized 3D printing and Mimics software for preoperative planning and intraoperative navigation in 21 cases of distal femoral fractures, allowing for accurate plate positioning and screw placement, as confirmed by postoperative CT. Arnal et al^[104]^ compared the use of 3D printed cutting guides with traditional techniques for opening-wedge distal femoral osteotomies, finding improved axial correction accuracy, reduced surgical time, and decreased fluoroscopy usage in the 3D printing group. Similarly, Shi et al and Chen et al^[114,115]^ demonstrated the benefits of 3D printed cutting and locking guides in medial closing-wedge distal femoral osteotomies for valgus knee malalignment.

Huang et al^[105,106]^ applied 3D printing technology to optimize screw placement in the management of tibial plateau fractures, achieving accurate fixation outcomes with minimal deviations between preoperative planning and postoperative screw trajectories. Giannetti et al^[116]^ compared the outcomes of minimally invasive reduction and internal fixation with and without 3D printing assistance in 40 cases of displaced tibial plateau fractures, demonstrating reduced surgical time, blood loss, and radiation exposure in the 3D printing group, with equivalent functional outcomes. Vaishya et al^[117]^ reported a case of a Schatzker type 2 proximal tibial fracture treated using a 3D printed model for fracture pattern delineation and precise implant placement. Yang et al^[118]^ investigated the use of 3D printing-assisted osteotomy for the treatment of malunited lateral tibial plateau fractures in 7 patients, facilitating accurate osteotomy planning and execution, reducing the risk of postoperative deformity, blood loss, and surgical time.

#### 4.2.3. Distal tibial and foot fractures

Distal tibial and foot fractures often present unique challenges due to their small size, complex anatomy, and limited soft tissue coverage. 3D printing has been employed to improve the understanding of fracture patterns, preoperative planning, and implant selection in these cases. Chung et al^[119]^ utilized 3D printing for understanding complex fracture patterns, preoperative templating, anatomical plate selection, and screw trajectory planning in the reduction and fixation of complex distal tibial fractures, achieving favorable results. Wu et al^[120]^ investigated the use of 3D printing techniques to determine optimal posterior screw placement and safe zones for screw fixation in talar neck fractures, potentially enhancing stability, reducing surgical time, and minimizing complications. Chung et al^[107]^ employed 3D printing to create models of calcaneal fractures and preshaped calcaneal plates for percutaneous fixation. Wu et al^[108]^ evaluated the effectiveness of 3D printing-assisted percutaneous minimally invasive reduction and cannulated screw fixation for intraarticular calcaneal fractures in 19 feet, leading to significant improvements in Bohler and Gissane angles, with excellent to good functional outcomes in the majority of cases.

#### 4.2.4. Ligament reconstruction

In addition to fracture management, 3D printing has been explored for ligament reconstruction procedures in the lower limb. Sha et al^[109]^ studied the anatomical reconstruction of lateral ankle ligaments using patient-specific navigational templates created by 3D printing in 15 cases of chronic ankle instability, facilitating precise and safe reconstruction of the lateral ligaments. Rankin et al^[121]^ designed a patient-specific, arthroscopic anterior cruciate ligament femoral tunnel guide for anatomical graft positioning based on MRI scans of the patient’s uninjured contralateral knee, demonstrating accurate replication of the anterior cruciate ligament femoral footprint size and position.

In conclusion, the application of 3D printing technology in lower limb trauma has yielded promising results in improving surgical planning, accuracy, and outcomes. By creating patient-specific models, guides, and implants, surgeons can better understand complex fracture patterns, optimize implant placement, and minimize complications. As the technology continues to evolve, it is anticipated to play an increasingly crucial role in the management of lower limb trauma, ultimately benefiting both surgeons and patients alike.

### 4.3. Pelvic and spinal trauma

The application of 3D printing technology in pelvic and spinal trauma has shown promising results, with several studies demonstrating its benefits in treating complex pelvic and acetabular fractures. Papotto et al^[12]^ conducted a systematic review revealing that 3D printing-assisted surgery for acetabular fractures resulted in shorter operative times, reduced blood loss, improved fracture reduction quality, and better clinical outcomes compared to traditional techniques. These findings are further supported by Tomaževič et al,^[13]^ who demonstrated in an experimental study that patient-specific 3D printed plates and drill guides significantly improved the accuracy of reduction in acetabular fracture models, showing smaller displacement of fracture lines compared to standard implants.

Beyond improving surgical outcomes, 3D printing technology has shown potential in reducing preoperative preparation time. Xu et al^[101]^ evaluated a novel method using 3D-printed plate templates for anterior pelvic fracture surgeries, demonstrating a significant reduction in plate pre-contouring time (by 93%) and 3D printing time (by 90%) compared to traditional methods. This time-saving aspect is particularly valuable in trauma settings where rapid intervention is often necessary. Moreover, Zhang et al^[102]^ reported that patients treated with 3D-printed custom-made metal plates for posterior wall and column acetabular fractures showed significantly better hip joint function and pain scores at 12 months postoperatively compared to those treated with traditional methods, suggesting long-term benefits of this technology.

While the advantages of 3D printing in pelvic trauma surgery are evident, its impact on postoperative recovery and hospital stay remains a subject of investigation. Hung et al^[122]^ found no significant difference in the length of hospital stay or ICU stay between 3D printing-assisted and conventional surgery groups in elderly patients with acetabular or pelvic fractures. However, their study highlighted the complexity of postoperative recovery in older adults and emphasized the need for comprehensive preoperative evaluations and personalized postoperative care plans.^[122]^ As 3D printing technology continues to evolve, it is likely to play an increasingly important role in the management of complex pelvic and spinal injuries, offering personalized solutions for challenging trauma cases while necessitating further research to fully understand its impact on postoperative recovery, especially in older patients.

### 4.4. Cost-effectiveness and implementation barriers

While 3D printing technology has demonstrated significant clinical benefits in orthopedic trauma, its widespread adoption faces various challenges related to cost-effectiveness and implementation barriers. The initial investment in 3D printing equipment ranges from $20,000 for basic FDM printers to over $500,000 for advanced metal printing systems.^[18]^ Additionally, ongoing costs include materials, maintenance, and specialized personnel training.^[3]^ However, several studies have shown potential cost savings through reduced operative time, decreased revision rates, and improved surgical outcomes. For instance, Hsu et al^[123]^ reported significant reductions in operative time and blood loss when using 3D-printed surgical guides for complex acetabular fractures, suggesting cost savings. The implementation of 3D printing technology in clinical practice requires consideration of various factors, including regulatory compliance, quality control protocols, and workflow integration.^[124]^ Healthcare institutions must develop standardized procedures for image acquisition, segmentation, design approval, and printing validation. Furthermore, the learning curve for surgical teams and technical staff represents a significant challenge that requires dedicated training programs and ongoing support.^[125]^ Despite these challenges, the long-term benefits of 3D printing technology may outweigh the initial investments, particularly in high-volume centers treating complex trauma cases.^[126]^ Table 4 summarizes the key aspects of cost-effectiveness and implementation barriers in orthopedic trauma applications.

**Table 4:** Cost-effectiveness analysis and implementation barriers of 3d printing in orthopedic trauma

Category	Component	Details	Impact/solutions
Initial Costs	Equipment	• FDM printers: $20,000–50,000	ROI feasible in high-volume centers
		• Metal printers: $300,000–500,000+	
	Software	• Imaging/CAD: $5000–20,000/year	Use open-source options initially
Ongoing Costs	Materials	• Standard: $50–200/kg	Negotiate bulk pricing
		• Medical-grade: $200–1000/kg	
	Personnel	• Training: $5000–15,000/person	Cross-train staff
	Maintenance	• 10–15% of equipment cost/year	Preventive maintenance
Cost Savings	Operating Room	• 20–30% reduced surgical time	Lower OR costs
	Clinical Outcomes	• 15–25% lower revision rates	Reduced readmissions
Implementation Barriers	Technical	• Learning curve for teams	Structured training programs
	Regulatory	• FDA/CE compliance	Regulatory consultation
	Workflow	• Integration into clinical practice	Standardized protocols

## 5. Conclusions and future perspectives

This review has explored the current developments in 3D printing technology for orthopedic trauma, highlighting its transformative potential across various applications. We have examined the main 3D printing technologies, including vat photopolymerization, material extrusion, powder bed fusion, and sheet lamination, detailing their principles, advantages, and limitations. The review has also addressed the challenges in treating complex traumatic fractures and how 3D printing offers innovative solutions. Specifically, we have discussed the applications of 3D printing in upper limb trauma, lower limb trauma, and pelvic and spinal trauma. The evidence presented demonstrates that 3D printing technology enhances surgical planning, improves the accuracy of fracture reduction, reduces operative time and blood loss, and potentially leads to better clinical outcomes in various orthopedic trauma scenarios. However, limitations such as material strength variability, sterilization challenges, and the surgeon learning curve must be considered to ensure safe and effective clinical adoption.

Looking ahead, the integration of 3D printing in orthopedic trauma is poised for significant advancements. Future developments are likely to focus on bioprinting technologies, enabling the creation of patient-specific, biocompatible implants with optimized mechanical properties and enhanced osseointegration. For instance, bioprinting could allow for the fabrication of living tissue scaffolds that promote bone regeneration, tailored to individual defect sites. The convergence of 3D printing with artificial intelligence and machine learning algorithms may revolutionize preoperative planning, allowing for automated design of patient-specific implants and surgical guides. AI could further optimize implant designs by predicting stress distribution based on patient-specific biomechanics, improving durability and fit. Additionally, the incorporation of smart materials and embedded sensors in 3D printed implants could facilitate real-time monitoring of healing processes and early detection of complications. Smart materials, such as shape-memory polymers, might adapt to postoperative changes, while sensors could track infection markers or load-bearing capacity. Emerging 4D printing, which introduces time-responsive adaptability, could enable implants to adjust dynamically to healing progression, as explored by Peng et al^[127]^ As the technology matures, we anticipate a shift towards point-of-care manufacturing, enabling on-demand production of custom implants in hospital settings. However, realizing these potential advancements will require addressing challenges such as regulatory approval processes, cost-effectiveness, and the need for large-scale clinical trials to validate long-term outcomes. The future of 3D printing in orthopedic trauma holds promise for increasingly personalized, efficient, and effective patient care, potentially transforming the field of orthopedic surgery.

## Acknowledgments

We would like to take this opportunity to express our sincere gratitude to West China Hospital, Sichuan University, for their strong support of this research. Figure 1 to 4 was created using BioRender.com (accessed on June 21, 2024).

## Author contributions

**Conceptualization:** Kun Ling, Jie Liu.

**Data curation:** Kun Ling.

**Formal analysis:** Kun Ling, Jie Liu.

**Funding acquisition:** Kun Ling.

**Investigation:** Kun Ling, Wenzhu Wang, Jie Liu.

**Methodology:** Kun Ling, Wenzhu Wang, Jie Liu.

**Project administration:** Kun Ling, Wenzhu Wang.

**Software:** Kun Ling, Wenzhu Wang.

**Supervision:** Kun Ling.

**Writing – original draft:** Kun Ling, Wenzhu Wang, Jie Liu.

**Writing – review & editing:** Kun Ling, Wenzhu Wang, Jie Liu.
